# Supplementary material for: How grid reinforcement costs differ by the income of electric vehicle users
Source: Nat Commun. 2024 Nov 8;15:9674. doi: 10.1038/s41467-024-53644-0 (PMC11549416; doi:10.1038/s41467-024-53644-0)
Supplement: Supplementary file 1 — Supplementary Information [file 41467_2024_53644_MOESM1_ESM.pdf]

Supplementary information: How grid reinforcement costs differ by the income of electric vehicle users

Sarah A. Steinbach[1*] and Maximilian J. Blaschke[1,2]

[1]Chair of Management Accounting, TUM School of Management, Arcisstraße 21, Munich, 80333, Germany.
[2]Center for Energy and Environmental Policy Research, Massachusetts Institute of Technology, 77 Massachusetts Avenue, Building E19-411, Cambridge, MA 02139-4307, USA.

*Corresponding author(s). E-mail(s): sarah.steinbach@tum.de; Contributing authors: maximilian.blaschke@tum.de;

Table of Contents

- Supplementary Table 1: Average distribution of household size per area type in Bavaria, Germany.
- Supplementary Table 2: Average distribution of households per building in Bavaria, Germany.
- Supplementary Figure 1: Breakdown of grid reinforcement costs asymmetries for all area types.
- Supplementary References

Supplementary Table 1: Average distribution of household size per area type in Bavaria, Germany.[1]

Persons per household	Rural	Suburban	Urban
1	35%	40%	54%
2	35%	33%	27%
3	14%	12%	10%
4	12%	11%	7%
5 or more	4%	4%	2%

Supplementary Table 2: Average distribution of households per building in Bavaria, Germany.[2]

Households per building	Rural	Suburban	Urban
1	70%	55%	53%
2	17%	13%	10%
3-6	9%	16%	14%
7-12	3%	12%	15%
13 or more	1%	4%	8%

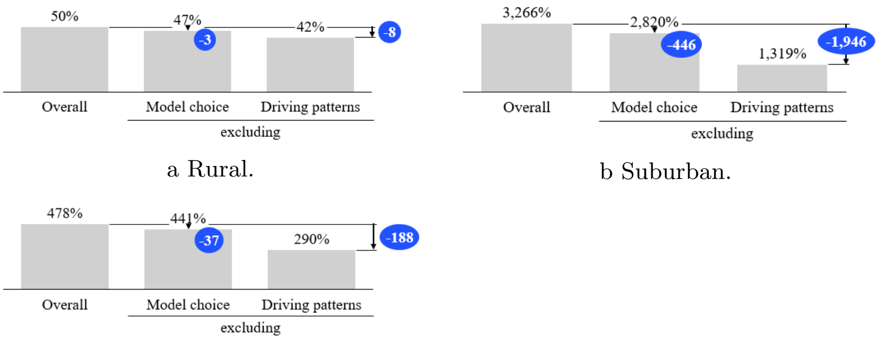

a Rural.

b Suburban.

c Urban.

Supplementary Figure 1: Breakdown of grid reinforcement costs asymmetries for all area types. We break down the difference in grid reinforcement costs between lower- and higher-income neighborhoods by the underlying drivers of model choice and driving patterns for the a rural, b suburban and c urban grid. The grey bars indicate the persisting cost difference. In blue, the respective effect of each driver is given. Source data are provided as a Source Data file.

Supplementary References

[1] Bayrisches Landesamt für Statistik. A6201c 201900 strukturdaten der bevölkerung und der haushalte in bayern teil i 2019. 5.2.

privathaushalte in bayern 2019 nach geschlecht des haupteinkommensbeziehers, gemeindegrößenklassen sowie haushaltsgröße (2021). URL https://www.statistik.bayern.de/statistik/gebiet_bevoelkerung/mikrozensus/index.html#link_1.

[2] Statistisches Bundesamt. Zensus datenbank (2021). URL https://ergebnisse2011.zensus2022.de/datenbank/online?operation=previous&levelindex=2&step=0&titel=Gebude+%28Tabellen%29&levelid=1645722017862&levelid=1645721940144#abreadcrumb.
